# Supplementary material for: Glucose Metabolism Disorders and Parkinson’s Disease: Coincidence or Indicator of Dysautonomia?
Source: Healthcare (Basel). 2024 Dec 6;12(23):2462. doi: 10.3390/healthcare12232462 (PMC11641510; doi:10.3390/healthcare12232462)
Supplement: Supplementary file 1 [file healthcare-12-02462-s001.zip › Table S1.pdf]

Table S1. Group characteristics and occurrence of autonomic disorders in patients with PD.

MDS-UPDRS p. III OFF, points [IQR]	51 [45-60]
MDS-UPDRS p. III ON points [IQR]	25 [20-32]
Hoehn–Yahr Scale points [IQR]	3 [2.5-3]
LEDD mg, [IQR]	1270 [790-2139.2]
SCOPA-AUT, points [IQR]	17.5 [8-28]
Autonomic symptoms, n %	
Gastrointestinal symptoms	8 (22.9)
Urinary dysfunction	12 (34.3)
Orthostatic hypotension	9 (25.7)
Hyperhidrosis	3 (8.6)

1. MDS-UPDRS—Movement Disorder Society Unified Parkinson’s Disease Rating Scale, LEDD—levodopa equivalent daily dose, SCOPA-AUT - The Scale for Outcomes in Parkinson's disease for Autonomic symptoms.
