# Supplementary material for: Glucose Metabolism Disorders and Parkinson’s Disease: Coincidence or Indicator of Dysautonomia?
Source: Healthcare (Basel). 2024 Dec 6;12(23):2462. doi: 10.3390/healthcare12232462 (PMC11641510; doi:10.3390/healthcare12232462)
Supplement: Supplementary file 1 [file healthcare-12-02462-s001.zip › Table S2.pdf]

Table S2. Characteristics of PD individuals with or without significant autonomic disorders.

	**Without autonomic disorders**	**With autonomic disorders**	**p**
Age, years	66 [55-72]	72 [68-79]	**0.0361**
Gender			0.2190
Male, %	10 (50)	5 (33.3)	
Female, %	10 (50)	10 (66.7)	
MDS-UPDRS p. III OFF	51 [45-58]	51 [49-64]	0.5268
MDS-UPDRS p. III ON	21 [18-30]	31 [23-34]	0.1171
Hoehn–Yahr Scale points	3 [2-3]	3 [2.5-3]	0.5611
Mass, kg	67.5 [62-90]	66.8 [53-77.6]	0.3217
Height, m	1.67 [1.6-1.7]	1.66 [1.58-1.74]	0.8596
BMI, kg/m^2	24.24 [22.5-25.2]	24.20 [22.4-25.1]	0.7657
LEDD	1231 [705-2139]	1230 [800-2520]	0.7321
Treatment:			
LCIG	4 (20)	3 (20)	0.9399
CSAI	2 (10)	2 (13.3)	0.6261
Oral Levodopa	15 (75)	9 (60)	0.2046
Dopamine agonists	13 (65)	9 (60)	0.4394
MAO-B Inhibitors	1 (5)	1 (6.7)	0.6952
COMT Inhibitors	4 (20)	3 (20)	0.9399
Amantadine	7 (35)	3 (20)	0.2466

1. Statistical analyses were performed using the Mann–Whitney U-test and Fisher's exact or chi-square tests. MDS-UPDRS—Movement Disorder Society Unified Parkinson's Disease Rating Scale; BMI—body mass index; LEDD—levodopa equivalent daily dose; LCIG – levodopa, carbidopa intrajejunal gel, CSAI – continuous subcutaneous apomorphine infusion; MAO-B - monoamine oxidase; COMT - catechol-O-methyltransferase.
