# Supplementary material for: Glucose Metabolism Disorders and Parkinson’s Disease: Coincidence or Indicator of Dysautonomia?
Source: Healthcare (Basel). 2024 Dec 6;12(23):2462. doi: 10.3390/healthcare12232462 (PMC11641510; doi:10.3390/healthcare12232462)
Supplement: Supplementary file 1 [file healthcare-12-02462-s001.zip › Table S3.pdf]

Table S3. Laboratory results in individuals with and without autonomic disorders.

	**Without autonomic disorders**	**With autonomic disorders**	**p**
Fasting glucose (mg/dL)	95.4 [91-101]	94.35 [87.4-110]	0.8386
HbA1C (%)	8.9 [6.1-16.4]	7.93 [5.6-16.9]	0.9018
Insulin (ng/dL)	189.5 [171-217]	164.5 [135-199]	0.1236
Total cholesterol (mg/dL)	112.8 [93-135]	80.7 [54.3-106]	**0.0089**
HDL cholesterol (mg/dL)	60.7 [44-70]	74.1 [61.6-79.7]	**0.0387**
LDL cholesterol (mg/dL)	82.4 [60.2-118]	63.9 [50.9-90.5]	0.1715
Triglycerides (mg/dL)	95.4 [91-101]	94.35 [87.4-110]	0.8386

Statistical analyses were performed using the Mann–Whitney U-test and Fisher's exact or chi-square tests.
